# Effects of soil on the development, survival, and oviposition of *Culex quinquefasciatus* (Diptera: Culicidae) mosquitoes

**DOI:** 10.1186/s13071-024-06202-y

**Published:** 2024-03-24

**Authors:** Kellen C. Pautzke, Allan S. Felsot, John P. Reganold, Jeb P. Owen

**Affiliations:** 1 Department of Entomology, College of Agricultural, Human, and Natural Resource Sciences, Washington State University, Pullman, WA USA; 2 Department of Crop and Soil Sciences, College of Agricultural, Human, and Natural Resource Sciences, Washington State University, Pullman, WA USA

**Keywords:** Southern house mosquito, Water chemistry, Larval survival, Oviposition, Behavior ecology

## Abstract

**Background:**

Water quality is known to influence the development and survival of larval mosquitoes, which affects mosquito-borne pathogen transmission as a function of the number of mosquitoes that reach adulthood and blood feed. Although water properties are known to affect mosquito development, few studies have investigated the link among soil properties, water quality, and mosquito development. Given the large number of ground-breeding mosquito species, this linkage is a potentially important factor to consider in mosquito ecology. In this study, we explored the effects of different soils on multiple life history parameters of the ground-breeding mosquito species *Culex quinquefasciatus* (Diptera: Culicidae).

**Methods:**

*Cx. quinquefasciatus* larvae were reared in water combined with different soil substrates (sandy, silt, or clay loam textures) at increasing soil to water volume ratios, with and without the addition of organic matter (fish food). Gravid mosquitoes were offered different soil–water extracts to investigate soil effects on oviposition preference.

**Results:**

Without the addition of organic matter, larval survival and development differed significantly among waters with different soil textures and volumes of substrate. Mosquitoes in water with clay loam soil survived longer and developed further than mosquitoes in other soil waters. Larvae survived for longer periods of time with increased volumes of soil substrate. Adding organic matter reduced the differences in larval survival time, development, and pupation among soil–water extracts. Adult female mosquitoes oviposited more frequently in water with clay loam soil, but the addition of organic matter reduced the soil effects on oviposition preference.

**Conclusions:**

This study suggests soil composition affects larval mosquito survival and development, as well as the oviposition preference of gravid females. Future studies could differentiate abiotic and biotic soil features that affect mosquitoes and incorporate soil variation at the landscape scale into models to predict mosquito population dynamics and mosquito-borne pathogen transmission.

**Graphical Abstract:**

**Figure:**
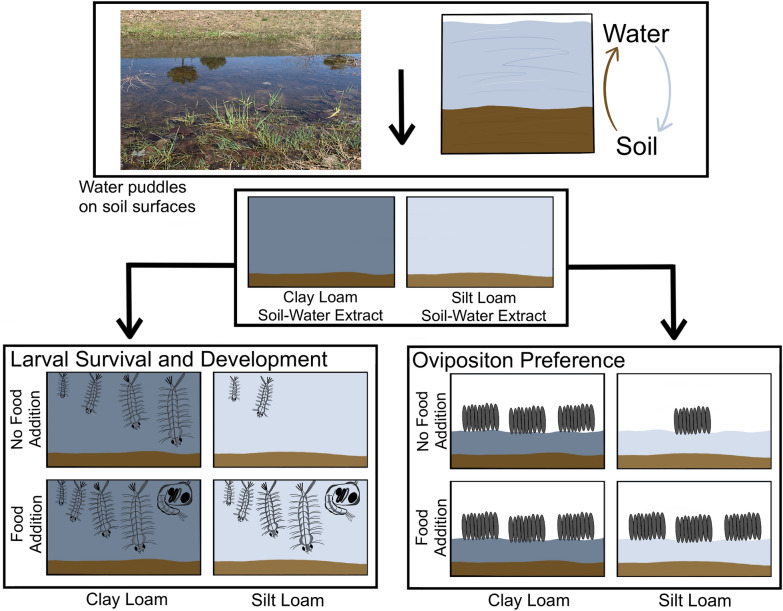

**Supplementary Information:**

The online version contains supplementary material available at 10.1186/s13071-024-06202-y.

## Background

Blood-feeding mosquitoes (Diptera: Culicidae) are the vectors of numerous human and animal pathogens throughout the world. These pathogens are the cause of over one million human deaths every year, with an estimated 80% of the global population living in areas that put them at risk of contracting one or more mosquito-borne pathogens [1, 2]. Variation in habitat can affect mosquito abundance and vector competence, underscoring the need to understand the relationships between environmental conditions and mosquito biology [3–6]. Furthermore, climate change and urbanization alter mosquito distribution and disease [7–9], which creates an imperative to accurately predict where mosquitoes are likely to develop and support mosquito borne pathogen transmission.

Juvenile mosquitoes are aquatic, requiring pools of standing water to develop. The quality of this aquatic habitat, which includes both physicochemical [10–12] and biological [12, 13] components, strongly affects larval development and survival. Assessments of larval habitats in both laboratory [10, 14, 15] and field settings [11, 16–20] have identified water parameters important to mosquitoes. Physicochemical metrics for larval habitat quality regularly include salinity [15, 18, 21], total dissolved solids (TDS) [22, 23], and pH [21]. Microorganisms in aquatic habitats provide nutritional resources for the larvae [13, 24] and are important for development into adulthood [25]. Adult female mosquitoes show preferences for oviposition sites based on visual and olfactory cues from the water that may reflect the quality of the aquatic habitat for larval development [26, 27]. Although female mosquitoes are known to exhibit preferences for oviposition sites, few studies have empirically investigated relationships between adult oviposition preferences and larval survival or development [28, 29].

Many mosquito genera, including *Culex* mosquitoes, are ground breeding and prefer pools of water that form on soil surfaces (ponds, puddles, floodplains, etc.) [30]. Characteristics of larval habitat can be modified by surface factors such as inflow of chemicals dissolved in runoff from agricultural or urban landscapes [31, 32], local vegetation [33], and biota living in or adjacent to the water [34]. Water also exchanges chemical and biological material with soil at the interfaces where it pools on the surface, and below ground in spaces that are created by soil aggregation [35]. Water parameters including salinity, total dissolved solids, and pH are modified through these interactions with soils [35]. Microorganisms, plant detritus, and animal-based organic matter from soils can also enter the water through this contact and contribute to the larval diet [13, 24]. Soils are chemically and biologically active and exhibit wide variation at different scales [35]. This variation is a product of multiple interconnected factors, including the mineral material, textural composition, historic and current plant and animal diversity, invertebrate and microbial communities, weather, and climate [35]. Soil texture, the proportion of differently sized mineral particles (sand, silt, and clay), affects the amount of organic matter, water and air flow, available water holding capacity, and microbial communities in a soil [35–39]. Thus, soil texture is a useful predictor for how a soil will interact with water.

Despite the well-known importance of water quality to mosquito life history and the interactions between water quality and soil, little is known about the relationships among all three [40, 41]. In this study, we examined the potential effects of several physically and chemically distinct soils on mosquito survival, development, and oviposition behavior. We approached the differences between soils holistically, treating biotic and abiotic features as integrated (i.e., soil reflects multiple components, including texture, minerals, organic matter, microbes, etc.). We tested the hypothesis that soils affect juvenile mosquito survival and development. Furthermore, we predicted that increasing amounts of organic matter in the soil would better support larval mosquito growth by providing nutritional resources for the larvae to consume. Lastly, we tested the hypothesis that soils influence adult mosquito oviposition behavior. Through these experiments, we aimed to broadly investigate the potential effects of soils on mosquito life history traits. Ultimately, soil may prove to be a useful parameter for predicting mosquito distribution and survival on a landscape.

## Methods

### Soil collection

Based on the United States Department of Agriculture (USDA) Natural Resources Conservation Service (NRCS) soil sample survey [42], we collected soil samples in eastern Washington State that represented three soil textures: sandy loam, silt loam, and clay loam. These textures reflect a range of soil particle sizes, including moderately coarse (sandy loam), medium (silt loam), and moderately fine (clay loam), which underpin differences in organic, mineral, and microbial compositions [35–39]. The collection sites were located on unmanaged land (sandy loam; 46.415328, −117.097164) and an active nature reserve (silt loam, clay loam; 46.728213, −117.139560) where the soils were not mechanically manipulated (e.g., tilling) or treated with chemicals (e.g., insecticides). We collected soils in several batches from June through September of 2019. At each collection site, we used a field texture test [43] to validate the soil texture before collection. Soil samples were later analyzed by Soiltest Farm Consultants (Soiltest Labs, Washington, USA) to confirm texture and composition [44]. At each site, we removed surface plants and large rocks before collecting soil samples with a post hole cutter (golf hole cutter) to obtain soil from the surface down to a depth of 15 cm [35, 45]. We collected a minimum of 30 cores from each site, with approximately 1 m separation between cores. For each soil texture, we combined samples in a large plastic tub and mixed them to homogenize the samples.

### Preparation of soil–water extracts

We dried all soils prior to use in experiments to remove residual water and allow soil macro-organisms (e.g., ants and earthworms) to escape. We transferred soils to large flat trays and left the soils outdoors in a shaded area to air-dry over 3 days (temperatures 24–30 ℃). Large aggregates were broken up to allow for even drying. This drying process paused biological activity [46] and made it so that all soil–water interactions in the experiment were controlled. No compositional modifications (microbial communities, mineral composition, etc.) were made to the soil substrates.

Prior to each experiment, we placed four liters of each dried soil substrate (unextracted) into separate 25 L rectangular tubs (16.5 × 33 × 30 cm). We combined each soil substrate with 4 L of double-distilled water by pouring the water along the side of the container to avoid disturbing the soil. We used double-distilled water so that there were no minerals in the water prior to soil extraction. We left the mixtures undisturbed for 7 days and suctioned out the soil-extracted water on day eight using a 50 mL pipette. We avoided collecting soil material with the water. We stored soil-extracted and control (double-distilled) waters in 15 L plastic containers until use. Using a mesh sieve (0.5 mm), we filtered the extracted waters before use in experiments. Minimal amounts of vegetation debris and soil sediment remained in the extracted waters. In all water samples, we measured water pH, salinity, total dissolved solids, and conductivity using an EC500 Waterproof ExStik II meter (FLIR Systems: Extech Instruments, New Hampshire, USA). We measured water properties at the beginning and end of each experiment.

### Mosquitoes

*Culex quinquefasciatus* mosquitoes used in these experiments were derived from a pathogen-free colony (JHB strain) established in the year 2000 by the Centers for Disease Control and Prevention (Atlanta, Georgia, USA). To maintain the colony, we reared hatched larvae in metal pans (15 × 30 cm) filled with double-distilled water kept in environmentally controlled enclosures (temperature: 25 °C ± 3; relative humidity (RH): 50% ± 10%; light cycle: 12/12). We fed larvae finely ground fish food (Kaytee Koi’s Choice, Chilton, WI, USA) until pupation and then placed pupae in screened insect enclosures (5800 cm^3^) to emerge. We provided females a human blood meal 4–6 days after emergence. Five days after blood feeding, we placed 5 oz opaque plastic cups containing 100 mL of double-distilled water in the adult mosquito cages. Gravid females oviposited in the water over a 12 h period (overnight). We then moved egg rafts to water-saturated filter paper to keep them moist and minimize trauma to the eggs during sorting for experiments. We separated the egg rafts using a 00-size artificial hair paintbrush and transferred 20 eggs (randomized from multiple egg rafts) to the surface of the water in each experimental cup (see below).

### Experiment 1: effects of soil on larval development and survival

To examine the effects of soil on larval survival and development, we reared larvae in water extracts from three different soil textures (clay loam, silt loam, sandy loam) combined with matching soil substrates at varying volumes. Experimental cups were first filled with a volume of fresh (unextracted) soil (0.1 mL, 1 mL, 10 mL, 25 mL, or 50 mL) and we then added soil-extracted water matching the soil substrate (see “Preparation of soil–water extracts” section above), so each cup contained 100 mL total of substrate and water. We placed 20 mosquito eggs in each experimental cup (9 cups per soil volume treatment) and stored the cups in an environmentally controlled tent (temperature: 25 ℃ ± 3; RH: 50% ± 10%; light cycle: 12/12). We added double-distilled water to the 100 mL line on each cup daily to counteract evaporation and keep relative solute concentrations constant. Following eclosion of first instar (L1) larvae in the cups, we recorded daily counts of living larvae, dead larvae, shed exoskeletons (evidence of development), and pupae [47, 48]. Based on the development time of our colony mosquitoes (10 days egg to adult), we terminated experiments on day 20 (colony development time + 10 days). Our rationale was that any mosquitoes not reaching adulthood by 20 days had stalled development and would fail to develop further. The experiment was replicated three times.

### Experiment 2: effects of added organic matter on larval development and survival

Based on results of experiment 1, we hypothesized that soil organic matter was insufficient to support larval survival and development. To test this hypothesis, we supplemented soil–water extracts with organic matter and compared larval survival and development among three conditions: (i) water extract + soil, (ii) water extract + supplemented organic matter, (iii) water extract + soil + supplemented organic matter. We used finely ground and homogenized fish food (Kaytee Koi’s Choice, Chilton, WI, USA) for all supplemental organic matter additions. Using pilot tests, we determined approximately 0.01 mg of finely ground fish food per larvae per development stage was sufficient to support development to pupation. We filled experimental cups with 50 mL of each soil and added 50 mL of the matching water extract, so each cup contained 100 mL total of soil substrate and water. We then supplemented the water in the cups with 0.2 mg total of fish food (20 larvae per cup × 0.01 mg food), based on pilot data. We provided 0.1 mg of fish food prior to egg placement, and a second addition of 0.1 mg on day seven. Control cups were filled with 100 mL of extracted soil water only. We stocked each cup (5 cups/treatment) with 20 mosquito eggs. The experiment was replicated two times.

### Experiment 3: effects of soil on adult oviposition behavior

To test for an effect of soil substrate on oviposition preference, we provided gravid females with cups containing different soil substrates and water. Using cages of 30–40 female mosquitoes (and approximately equal numbers of males), we offered human blood meals 4 days after eclosion. On the day of blood feeding, we prepared four water cups for each adult mosquito cage. We used black plastic cups to reduce any potential effect of water color on oviposition preference [49–52]. Three cups contained 30 mL of soil and 30 mL double-distilled water, with one soil texture per cup (i.e., each cup contained a unique soil texture). We tested the same textures used in the larval development experiments (clay loam, silt loam, and sandy loam). We filled a fourth cup with only double-distilled water as a positive control, given that double-distilled water was used as the only oviposition site for our colony mosquitoes. Five days after preparing the cups, we measured water parameters in each cup using an EC500 Waterproof ExStik II meter. We placed the four unique cups into each mosquito cage, randomly assigning a cup to each of the four corners of the cage so that there was no position bias among replicates. We left the cups in the cages for 12 h (overnight) for oviposition. After 12 h the egg rafts in each cup were counted and recorded. We repeated this experiment 20 times, using a different cohort of mosquitoes and new soil–water cups for each replicate.

To examine how added organic matter might alter the effects of soil on mosquito oviposition behavior, we repeated the experiment with the addition of a fixed quantity of organic matter in the oviposition cups. We prepared the experimental cups as described above but added 0.1 mg of finely ground fish food (Koi’s Choice) to each cup, including the double-distilled water control. We placed the four types of cups into each cage with random assignment of one cup to each corner of the cage. We left the cups for 12 h (overnight) for oviposition. Upon cup removal, we counted and recorded the egg rafts in each cup. We repeated this experiment six times, using a different cohort of mosquitoes and new soil–water cups for each replicate.

### Statistical analyses

All statistical analyses were performed in R (version 3.6.3, RStudio version 1.2.5033) [53]. We used daily counts of larval survival to determine survival times per cup. We treated the time point where 50% of the larvae in a cup had died as median survival time. We used generalized linear mixed effects regression models with a Poisson distribution to determine what variables affected median survival times while controlling for any replicate effect [54]. For experiment 1, we used the variables soil texture, soil volume, and experimental replicate (as a random effect) to model median survival time. For experiment 2, we used the variables soil texture, organic supplementation (yes/no), and experimental replicate (as a random effect) to model median survival time. Larval development data were determined from counts of individuals reaching each developmental instar (stage L1–L4). Larval development counts were analyzed using Chi-square tests to examine the effects of soil substrate, soil volume, and organic matter addition. Water measurements including salinity, total dissolved solids, and pH, were compared using one-way analysis of variance (ANOVA) tests. Counts of egg rafts laid by gravid females (adult oviposition preference) were compared among different soils using generalized linear mixed effects regression models [54].

## Results

### Soil analysis

Each of the three soil textures had distinct soil properties (Table 1). The pH values of the soils ranged from 6.6 in silt loam to 7.2 in both sandy loam and clay loam (Table 1, 1:1 pH column). The silt loam soil had the highest salt concentrations [electrical conductivity] (EC = 0.936), and the sandy loam had the lowest salt level (EC = 0.745). The percent organic matter was 1.9% in sandy loam, 3.5% in silt loam, and 4.5% in clay loam (Table 1, OM column). Given that soil physicochemical properties varied as a function of soil texture, the term soil texture will henceforth be used as a holistic descriptor to differentiate the soils.

**Table 1:** Soil analysis results from Soiltest Farm Consultants. EC, electrical conductivity; NO_3_-N, NH_4_-N, Olson P, K, Ca, Mg, Na are elements and chemical compounds denoted by their chemical symbols; OM, organic matter

Variables	1:1 pH	EC	NO_3_-N	NH_4_-N	Olson P	K	Ca	Mg	Na	OM
Soil		mmhos/cm	mg/kg	mg/kg	mg/kg	mg/kg	meq/100 g	meq/100 g	meq/100 g	%
Sandy loam	7.2	0.754	2.9	1.8	23	429	10.1	2	0.15	1.9
Silt loam	6.6	0.936	7.8	3	40	640	13.7	4.1	0.14	3.5
Clay loam	7.2	0.806	1.1	1.8	38	413	16.9	6.1	0.36	4.5

### Effects of soil on water chemistry

**Fig. 1:**
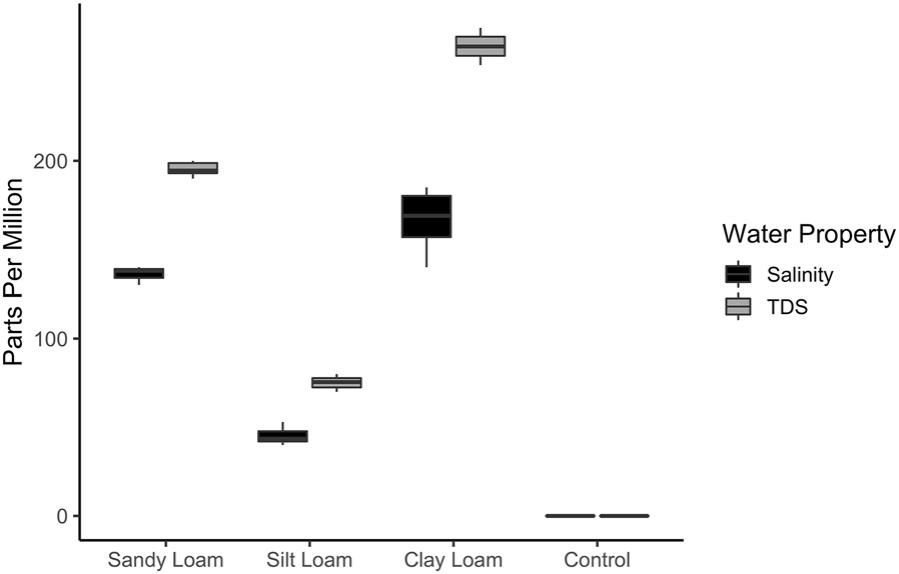
Average soil–water extract values (salinity and total dissolved solids) in parts per million (*y* axis; ppm). Measurements from each soil–water extract across all experimental replicates were pooled. The boxes show the interquartile range of water property values and the median is shown as a solid black line. Control (double-distilled water) values were 0

Contact with soil substrates altered the physicochemical properties of water, as demonstrated by the differences in water quality values between double-distilled water and the extracted soil waters (Fig. 1; Additional file 1: Table S1). Both salinity (one-way ANOVA, *F*_(2,51)_ = 823.6, *P* < 0.0001) and TDS (*F*_(2,51)_ = 7606, *P* < 0.0001) were significantly different across the three soil–water extracts. The average (± standard deviation, SD) salinity value was highest for clay loam water (166.0 ± 15.3) compared with sandy loam (136.6 ± 3.2) and silt loam (44.9 ± 4.2) waters. The average TDS value was highest for clay loam water (264.4 ± 6.7) compared with sandy loam (195.4 ± 3.3) and silt loam (75.2 ± 3.1) waters. The measured water properties remained at equilibrium for the three soil textures over the duration of the experiments (Additional file 2: Fig. S1).

### Experiment 1: effects of soil on larval development and survival

**Fig. 2:**
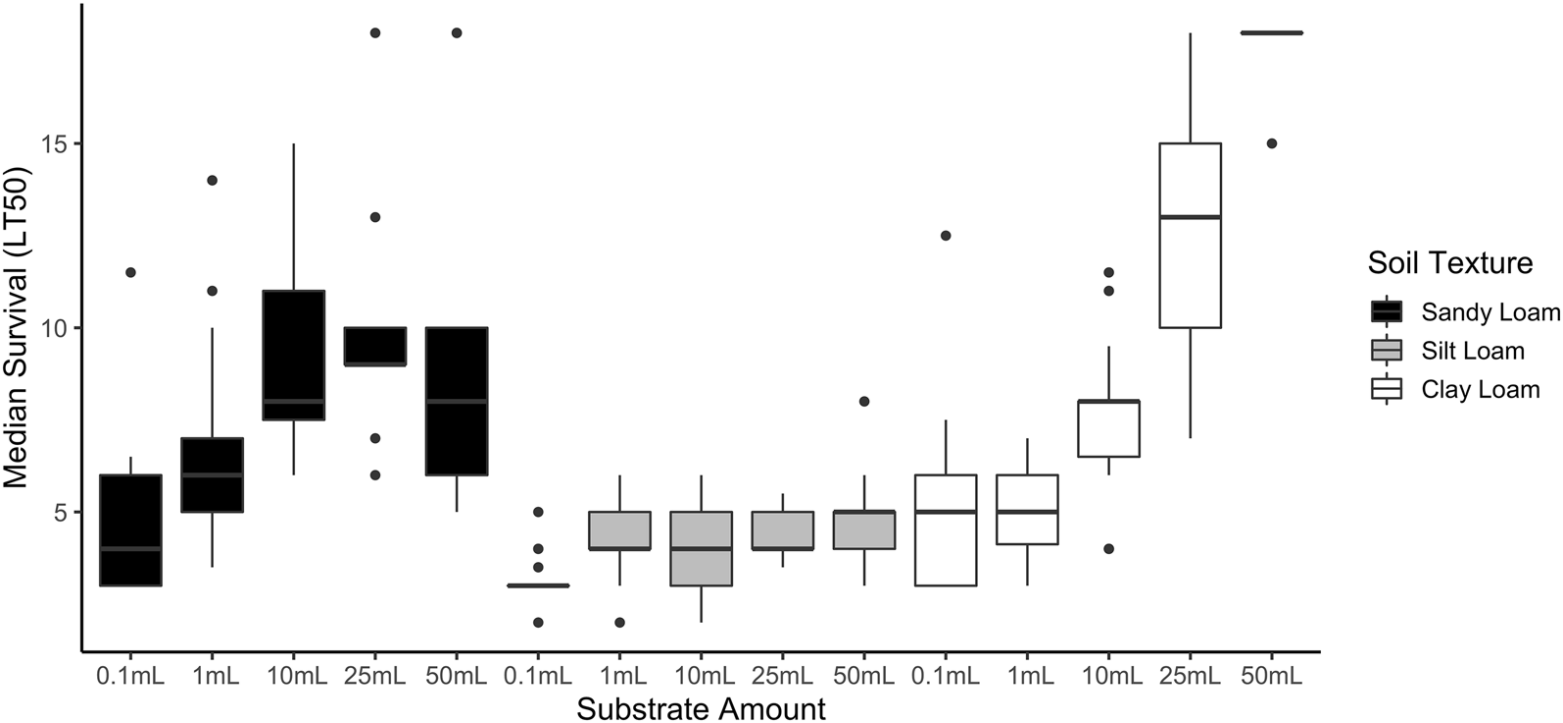
Larval survival in different soil volumes and textures. Median survival times (LT50; *y* axis) are shown for larvae in soil–waters with different soil volumes (0.1 mL, 1 mL, 10 mL, 25 mL, 50 mL) and soil textures: sandy loam (black boxes), silt loam (gray boxes), and clay loam (open boxes). The boxes show the interquartile range of median survival times, the median is shown as a solid black line, and outliers as black circles

There were significant differences in larval survival times, due to soil texture and volume (Fig. 2). Larvae survived for longer periods of time in cups with clay loam (average median survival time = 7.64 days) and sandy loam (7.35 days) soils than in silt loam soils (4.02 days) (mixed effects regression, *t*_(232)_ = −2.20, *P* = 0.028). Cups with more soil substrate supported longer larval survival, with better survival at the 25 mL (*t*_(232)_ = 4.990, *P* < 0.0001), and 50 mL volumes (*t*_(232)_ = 4.647, *P* < 0.0001). Overall, larvae in clay loam water lived the longest (median survival range = 4–18 days) and larvae in silt loam lived the shortest (median survival range = 3–5 days) across all soil volumes.

Development of larvae was affected by both soil texture (Chi-square test, *χ*^*2*^ = 457.4, *df* = 6, *P* < 0.0001) and soil volume (*χ*^*2*^ = 1070.2, *df* = 12, *P* < 0.0001) (Additional file 3: Table S2). In silt loam water, < 2% of larvae developed past the L1 stage, and none developed past L2. In contrast, 35.0% and 39.0% of larvae developed past L1 in sandy loam and clay loam waters, respectively. Larvae developed the furthest in clay loam water, with 13.6% of larvae in clay loam water reaching the fourth larval instar (L4), compared with only 1.0% in sandy loam water and 0% in silt loam water.

### Experiment 2: effects of added organic matter on larval development and survival

**Fig. 3:**
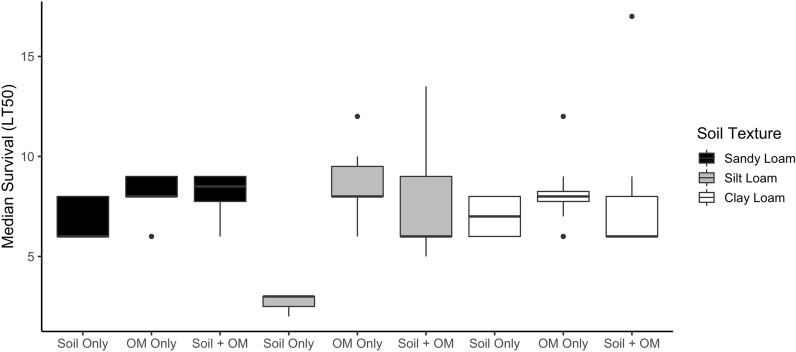
Larval survival with and without added organic matter. Median survival times (LT50; *y* axis) are shown for larvae in soil–water extracts combined with soil substrate only (soil only), organic matter only (OM only), or both soil substrate and organic matter (Soil + OM). The boxes show the interquartile range of median survival times, the median is shown as a solid black line, and outliers as black circles

The addition of organic matter improved larval survival and reduced differences in survival times associated with soil texture (Fig. 3). Larvae survived longer in cups with added organic matter than in cups with only soil (mixed effects Regression, *t*_(74)_ = −3.753, *P* = 0.0001). For cups with water and soil only, survival time was significantly shorter in silt loam (average median survival time = 2.78 days) than in sandy loam (6.86 days) and clay loam (7.0 days) (*t*_(74)_ = −3.778, *P* = 0.0001). In cups with added organic matter, there was no difference in survival times among soil textures (*t*_(74)_ = 0.075, *P* = 0.944).

Larval development was affected by soil texture (*χ*^*2*^ = 252.6, *df* = 8, *P* < 0.0001) and the addition of organic matter (*χ*^*2*^ = 50.8, *df* = 4, *P* < 0.0001) (Additional file 3: Table S2). For cups with added organic matter, approximately 24% of larvae developed past the first larval instar (L1) in silt loam waters. In contrast, 55% and 81% larvae developed past L1 in sandy loam and clay loam waters, respectively. In all soils with added organic matter, some larvae reached pupation, compared with no pupation in the cups with no organic matter added. In clay loam water, approximately 59% of larvae reached the fourth larval instar (L4) compared with only 14% in sandy loam water and 9% in silt loam water. The numbers of pupae were similar between the cups with added organic matter, with each soil texture averaging 13 total pupae.

### Experiment 3: effects of soil on adult oviposition behavior

**Fig. 4:**
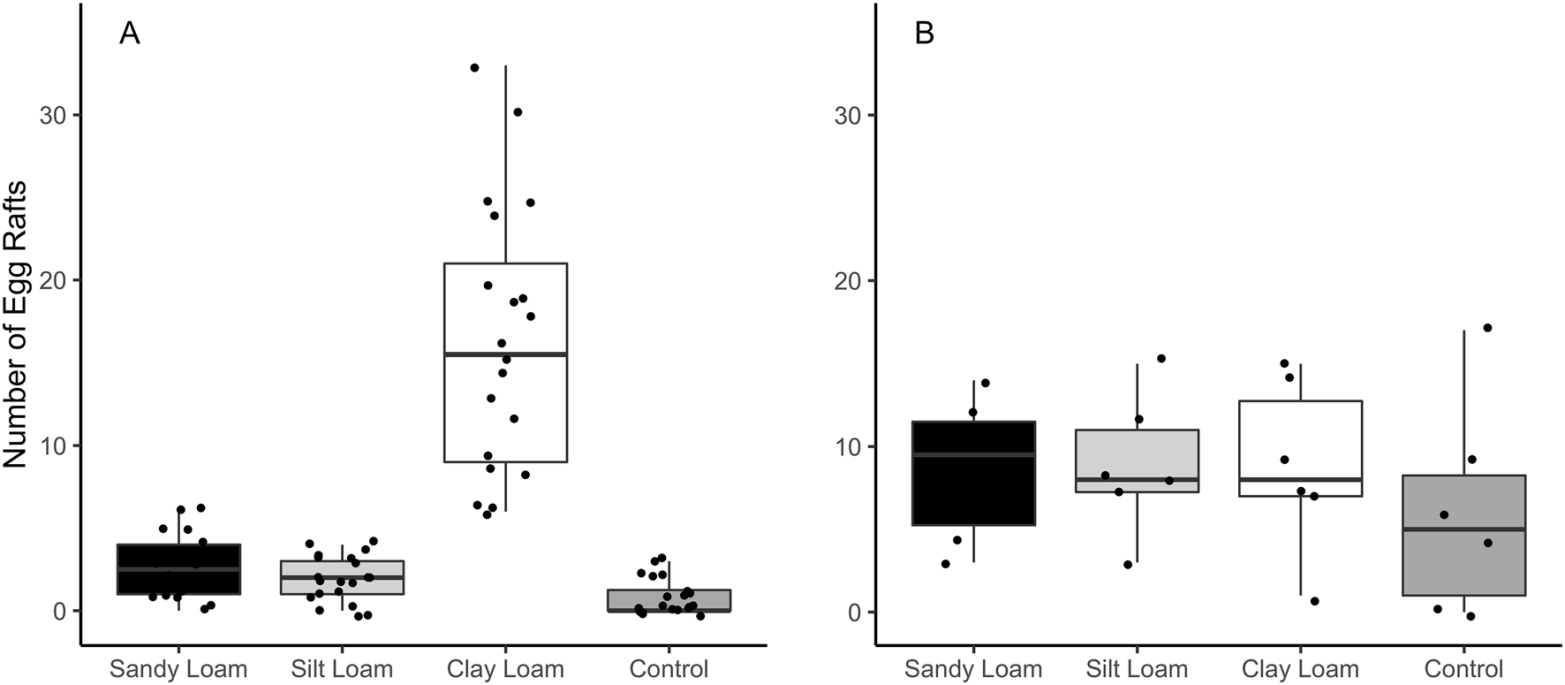
Effects of soil texture on oviposition. Numbers of egg rafts (*y* axis) laid in water cups are shown for different treatments (*x* axis) from experiments with **A** only soil–water and soil substrate (no organic matter added) and **B** soil–water, soil substrate, and 0.1 mg added organic matter (fish food). Individual plot points denote the number of egg rafts oviposited in each treatment cup in a single experimental replicate. The boxes show the interquartile range of egg raft counts, the median is shown as a solid black line, and outliers as black circles

Oviposition was significantly different among soil textures, when no supplemental organic matter was added (mixed effects regression, *t*_(80)_ = −11.78, *P* < 0.0001). Gravid female mosquitoes preferentially oviposited in waters with clay loam soils, which accumulated approximately 75% of all egg rafts (Fig. 4A). When organic matter was added to the water, the effect of soil texture on oviposition preference was reduced (Fig. 4B). Egg raft count did not differ significantly between the three soil substrates and double-distilled water control when organic matter was added (*t*_(24)_ = 0, *P* = 1).

## Discussion

The physicochemical and biological properties of the aquatic habitat are known to affect survival and development of mosquito larvae [10–25], but relatively little is known about effects of soil on these interactions for ground-breeding mosquitoes [40, 41]. We observed that soil texture, soil volume, and organic matter inputs all affected the survival and development of larval *Cx. quinquefasciatus* mosquitoes. Overall, clay loam and sandy loam soil waters supported longer survival and further development of larvae when compared with silt loam water. The addition of organic matter (fish food) to the water improved survival and development in all soil waters, but it did not eliminate soil-mediated effects. Even with the addition of organic matter, most larvae in silt loam water did not develop as far as larvae in sandy and clay loam waters. Soils also affected mosquito oviposition behavior. Gravid females displayed a strong preference for clay loam water for oviposition. However, oviposition preference disappeared after organic matter was added to the different waters, suggesting that organic matter inputs strongly affect the perceived quality of the larval habitat [26, 27, 29].

We predicted that soils with increasing amounts of soil organic matter (SOM) would better support larval mosquito growth [17, 23, 24, 33, 34]. Our data did not support that prediction. Silt loam had a higher percent of SOM compared with sandy loam (3.7% versus 1.9%, respectively), but larval survival and development were significantly better in the sandy loam water. These results could be due to differences in SOM characteristics, including the composition or nutrient bioavailability [55, 56]. Additionally, variation in SOM composition may have resulted in differences in soil microbial communities that likely constituted part of the larval diet [13, 24]. Okech et al. [40] examined how naturally occurring variation in soil organic matter affected larval *Anopheles* spp. mosquito life history traits, finding that adult size and vector competence were positively correlated with increasing percent SOM. Manipulating the original microbial communities by autoclaving the soils altered these relationships; however, the microbial composition before and after autoclaving was not described. Pfaehler et al. [41] also investigated relationships between naturally occurring variation in SOM and *Anopheles* spp. mosquitoes. They observed negative correlations between SOM and development time and adult size, and a positive correlation between SOM and pupation rate. The contrasting results of these studies suggest that mechanisms of interaction between SOM and mosquito development are complex and may depend on the nature of the organic matter [33, 34, 55, 56], interactions between SOM and the microbial community [36, 38, 39], and involvement of abiotic water parameters [35].

While the percent organic matter in the soil was not correlated with larval survival or development in our experiments, we did observe positive effects of adding organic matter to the water [13, 20, 28, 29]. The addition of organic matter improved larval survival and development across all soil substrates, reducing soil-mediated effects most notably for larvae in silt loam water. With the soil substrate alone, larvae in silt loam water did not develop beyond the second instar. In contrast, the addition of organic matter to silt loam water supported development of some larvae to adulthood. Despite this, differences in development remained among soil substrates after organic matter addition. Higher numbers of larvae developed further in clay loam water compared with silt loam water. These results underscore that the quality of the larval habitat is not solely dependent on organic matter availability but is a product of interactions between water chemistry, soil composition, and organic matter [18, 23, 24, 34].

Our study suggests that water properties affected by soil (e.g., salinity, total dissolved solids, and conductivity) may have influenced mosquito survival and development. We observed that larval survival time and development increased as salt and TDS measures increased among the three soil–waters. Numerous field surveys of mosquito abundance have suggested that physicochemical water properties such as salinity and TDS are positively correlated with larval abundance [10–25]. Experimental studies across mosquito species, such as those done by Clark et al. [14] and Patrick et al. [15], have demonstrated relationships between salinity, larval development, and survival. Akpodiete et al. [10] experimentally demonstrated increased larval survival and shortened development time in waters with higher mineral content. Multini et al. [21] suggest that pH is one of the more important physicochemical parameters driving larval survival, citing abundance studies across multiple species. Total dissolved solids (TDS) are suggested to be important for larval survival by providing protection from solar radiation [22] and by providing nutritional resources to larval mosquitoes [23, 24]. Together, these studies suggest multiple mechanisms by which soil effects on water could influence mosquito survival and development.

In addition to effects on larvae, water quality is known to influence female mosquito oviposition [26, 27]. Initial selection of oviposition sites is based on several sensory cues including reflected or emitted light (infrared, ultraviolet) and volatile chemicals in the water originating from plants, microorganisms, other mosquitoes, and predators [26, 27, 57]. Before depositing eggs, gravid females also use water contact cues to further evaluate a site, including water surface temperature and chemical composition [26, 27]. Given our observations of differential survival and development between soils, we tested for oviposition preferences to determine whether there was alignment between oviposition and the relative quality of the larval habitat. We observed more frequent oviposition in clay loam water that best supported larval development and less oviposition in silt loam and double-distilled waters where larval survival was low. There were similar levels of oviposition in sandy loam water and silt loam water, despite higher survival in sandy loam water. The observed oviposition preferences could be due to volatile attractants in the different soils [58], mediated by biotic factors such as microorganisms, or the physicochemical properties of the different waters [57], mediated by chemical and organic characteristics of the soils. Herrera-Varela et al. [59] tested relationships between larval survival and adult oviposition in *Anopheles* spp. mosquitoes by providing gravid females choices between autoclaved or nonautoclaved soil–water, lake water, and hay-infused water. They found a significant relationship between oviposition rates and larval survival, with females preferentially selecting the nonautoclaved and infused waters that supported higher larval survival. Our initial oviposition experiment also suggested that females select waters that better support larval development. However, that preference was eliminated by the addition of organic matter to the water, even though clay loam water remained superior for development in our larval studies. These results suggest that perception of organic matter in the water may override female responses to other water quality cues. Given that water will contain materials derived from the soil and external inputs (e.g., vegetation), it is unclear how the results of our experiments may translate to mosquito behavior in the field. Additionally, the mechanisms driving this observed oviposition behavior are unknown, as our experimental design did not tease apart the potential biotic and abiotic features of the soils that could drive oviposition preferences. Nonetheless, our studies do suggest that soil properties could affect mosquito abundance as a function of larval survival, oviposition preference, or both.

Soils have been generally overlooked as a feature of mosquito ecology. Some studies have examined oviposition relative to soil vegetation, moisture, and color [60–64] and many studies have been conducted as remote sensing and modeling efforts, rather than manipulative experiments [65–67]. If we can develop a robust understanding of the relationships among mosquitoes, water, and soils, we can potentially use available fine-scale soil data to better predict mosquito distribution and survival on a landscape scale. This is particularly important given global changes in precipitation and temperatures are expected to affect the distributions and densities of mosquito populations [68–71]. For example, Samy et al. [72] used global temperature and precipitation data to predict future distributions of *Cx. quinquefasciatus* mosquitoes. Their models identified a broad increase in habitat suitability in southern Australia due to predicted changes in annual temperatures and precipitation. Although the climate modeling by Samy et al. [72] indicates a range shift is possible, our data suggest that soil variation could constrain the realized locations of range expansion for *Cx. quinquefasciatus*. The Commonwealth Scientific and Industrial Research Organisation (CSIRO) Soil and Landscape Grid of Australia [73] and the CSIRO National Soil Site database [74] provide insight into the variation of soil textures in Australia. Looking specifically in southern Australia, the amounts of clay in the soils ranges from less than 10% to more than 45%, and amounts of sand in soils ranges from 20% to more than 80% [73]. These variations in soil texture affect the potential chemical and biological characteristics of a soil [35, 37, 38]. Based on our study results, the soil variability in areas of Australia predicted to experience *Cx. quinquefasciatus* range expansion may alter the suitability of habitat for range expansion predicted by general climate models. While soils are susceptible to alterations brought on by climate change as well, they will remain an important feature of the landscape and retain the ability to influence mosquito habitat suitability [75]. From a global perspective, and with consideration of the biodiversity of mosquitoes, the actual locations of mosquito range expansion may reflect both soil effects as well as broad climate conditions. Identifying soil–water interactions that alter the quality of larval habitat is necessary to better predict global mosquito distribution and vector population dynamics.

The results of our study have several caveats. First, we treated soils holistically (i.e., a soil is a product of multiple interacting components, including texture, minerals, microbes, organic matter, etc.) and characterized only a subset of soil characteristics. Notably, we were unable to characterize the microbial communities of the soils, which may have affected nutritional resources for the larvae [13, 24]. Unfortunately, measuring the microbial community (such as by substrate induced respiration, microbial biomass, phospholipid fatty acid analysis, specific microbial enzyme activities, or different molecular techniques) was beyond the scope of this study. By selecting different soils based on texture, we predicted that the soils would likely have differences in microbial communities, mineral composition, and organic matter [35–39], but were only able to feasibly confirm the latter two. To better understand the mechanisms of soil–water–mosquito interactions, microbial analyses should be considered in future work. Other than the removal and addition of water, we did not manipulate components of the soil. As a result, it is unclear what exact properties of the soils may have affected mosquito survival, development, and behavior. The organic matter supplemented in the water was loosely reflective of organic matter inputs in the field, as typical larval diets include plant and animal detritus, and microorganisms [13, 24]. The bioavailability of the supplemented organic matter and its use by the larvae or microorganisms in the water may have differed from natural sources. Moreover, as our present study included only laboratory-controlled experiments, our findings were limited in scope and excluded potentially important ecological factors such as fluctuations in temperature, predation pressure, and competition. Despite these important limitations, our results do illustrate that soil can strongly affect mosquito survival, development, and behavior. Future work should endeavor to determine the underlying mechanisms of these effects, but also make use of the macro-scale importance of soil effects when soils are appreciated holistically as complex, interacting abiotic and biotic elements.

## Conclusions

The findings of this study show how soils, which are highly variable components of the environment, alter water properties and affect the life history traits of *Culex quinquefasciatus* mosquitoes. Soil texture and soil volume had significant effects on the survival times and development of larval mosquitoes. The addition of organic matter (fish food) significantly reduced the effect of soil substrate on larval survival times and development. Adults demonstrated strong oviposition preferences based on soil substrate, and the addition of organic matter eliminated those preferences. Overall, the composition of the soil component in mosquito habitat has the potential to influence the development, survival, and distribution of mosquitoes. Due to the variability of soil chemical composition, a finer-scale exploration of the relationships between soil and mosquitoes would strengthen our understanding of this environmental variable in mosquito ecology. Because the experiments performed in this study were conducted in a laboratory setting, future testing should explore how these results translate to mosquitoes in field conditions. Additionally, future studies should consider how soil microbial communities and soil organic matter composition affect the variation in soils in the context of mosquito life history traits.

### Supplementary Information

**Additional file 1: Table S1.** Average (± standard deviation, SD) water properties measured from soil-water extracts. Water quality measurements were taken from soil–water extracts from all experimental replicates and pooled. Control (double-distilled water) values were 0.

**Additional file 2: Figure S1.** Physicochemical measurements of soil–waters **A** before and **B** after each experiment. The measured values of salinity (black boxes) and total dissolved solids (gray boxes) in parts per million (*y* axis; ppm) are shown for each soil-water treatment across all experiments (*x* axis). The boxes show the interquartile range of water property values, the median is shown as a solid black line, and outliers as black circles.

**Additional file 3: Table S2. **Terminal development stage (highest developmental stage reached before death or experiment termination) are shown for larvae in experiments 1 and 2 by treatment. L1–L4 denote larval instars 1–4.

## Data Availability

All data generated or analyzed during this study are included in this published article.
