# Supplementary material for: Effects of soil on the development, survival, and oviposition of Culex quinquefasciatus (Diptera: Culicidae) mosquitoes
Source: Parasit Vectors. 2024 Mar 24;17:154. doi: 10.1186/s13071-024-06202-y (PMC10960989; doi:10.1186/s13071-024-06202-y)
Supplement: Supplementary file 1 — Additional file 1: Table S1. Average (± standard deviation, SD) water properties measured from soil-water extracts. Water quality measurements were taken from soil–water extracts from all experimental replicates and pooled. Control (double-distilled water) values were 0. [file 13071_2024_6202_MOESM1_ESM.docx]

| Soil | pH | Salinity (ppm) | Total Dissolved Solids (ppm) | Conductivity (μS) |
| --- | --- | --- | --- | --- |
| Sandy Loam | 7.2 (±0.09) | 136.6 (±3.2) | 195.4 (±3.3) | 273.1 (±7.1) |
| Silt Loam | 6.9 (±0.16) | 44.9 (±4.2) | 75.2 (±3.1) | 101.1 (±4.9) |
| Clay Loam | 7.2 (±0.10) | 166.0 (±15.3) | 264.4 (±6.6) | 364.6 (±13.2) |
| Double Distilled Water | 6.9 (±0.05) | 0 (±0) | 0 (±0) | 0 (±0) |
