# Supplementary material for: Effects of soil on the development, survival, and oviposition of Culex quinquefasciatus (Diptera: Culicidae) mosquitoes
Source: Parasit Vectors. 2024 Mar 24;17:154. doi: 10.1186/s13071-024-06202-y (PMC10960989; doi:10.1186/s13071-024-06202-y)
Supplement: Supplementary file 3 — Additional file 3: Table S2. Terminal development stage (highest developmental stage reached before death or experiment termination) are shown for larvae in experiments 1 and 2 by treatment. L1–L4 denote larval instars 1–4. [file 13071_2024_6202_MOESM3_ESM.docx]

| Experiment 1: Effects of soil on larval development and survival | | | | | |
| --- | --- | --- | --- | --- | --- |
|  | L1 | L2 | L3 | L4 |  |
| Sandy Loam 0.1 mL | 168 | 4 | 0 | 0 |  |
| Sandy Loam 1 mL | 179 | 13 | 0 | 0 |  |
| Sandy Loam 10 mL | 95 | 174 | 15 | 0 |  |
| Sandy Loam 25 mL | 14 | 36 | 20 | 0 |  |
| Sandy Loam 50 mL | 45 | 0 | 0 | 8 |  |
| Silt Loam 0.1 mL | 197 | 0 | 0 | 0 |  |
| Silt Loam 1 mL | 177 | 0 | 0 | 0 |  |
| Silt Loam 10 mL | 197 | 8 | 0 | 0 |  |
| Silt Loam 25 mL | 74 | 3 | 0 | 0 |  |
| Silt Loam 50 mL | 63 | 3 | 0 | 0 |  |
| Clay Loam 0.1 mL | 155 | 7 | 0 | 0 |  |
| Clay Loam 1 mL | 129 | 31 | 0 | 0 |  |
| Clay Loam 10 mL | 114 | 112 | 15 | 0 |  |
| Clay Loam 25 mL | 9 | 1 | 12 | 84 |  |
| Clay Loam 50 mL | 38 | 2 | 10 | 16 |  |
|  |  |  |  |  |  |

| Experiment 2: Effects of added organic matter on larval development and survival | | | | | |
| --- | --- | --- | --- | --- | --- |
|  | L1 | L2 | L3 | L4 | Pupa |
| Sandy Loam Soil Only | 24 | 4 | 8 | 15 | 0 |
| Sandy Loam Soil + Food | 29 | 4 | 37 | 14 | 10 |
| Sandy Loam Food Only | 73 | 16 | 19 | 19 | 6 |
| Silt Loam Soil Only | 69 | 4 | 3 | 0 | 0 |
| Silt Loam Soil + Food | 68 | 3 | 11 | 4 | 2 |
| Silt Loam Food Only | 76 | 2 | 2 | 13 | 8 |
| Clay Loam Soil Only | 14 | 10 | 7 | 13 | 0 |
| Clay Loam Soil + Food | 0 | 0 | 10 | 71 | 9 |
| Clay Loam Food Only | 40 | 7 | 15 | 51 | 9 |
